# Prevalence of co-infection and genetic diversity of avian haemosporidian parasites in two rehabilitation facilities in Iran: implications for the conservation of captive raptors

**DOI:** 10.1186/s12862-022-02068-9

**Published:** 2022-10-08

**Authors:** Leila Nourani, Mansour Aliabadian, Omid Mirshamsi, Navid Dinparast Djadid

**Affiliations:** 1 Malaria and Vector Research Group (MVRG), Biotechnology Research Center (BRC), Pasteur Institute of Iran, Tehran, Iran; 2 Department of Biology, Faculty of Science, Ferdowsi University of Mashhad, Mashhad, Iran; 3 Research Department of Zoological Innovations (RDZI), Institute of Applied Zoology, Faculty of Science, Ferdowsi University of Mashhad, Mashhad, Iran

**Keywords:** Birds of prey, Avian malaria, Vector-borne disease, Haemosporidian parasites

## Abstract

**Background:**

Various haemosporidian parasites infect raptors, especially captive hosts who may be more exposed. Diagnosis of threatening factors such as infectious diseases indirectly has a significant role in protecting endangered or threatened species that may boost the mortality or extinction resulting from declined reproduction. Few investigations have been performed in captive hosts to detect the prevalence of haemosporidian parasites and define genetic diversity in west Asia. For the first time, the current study was designed to determine the prevalence and genetic diversity of haemosporidian parasites in captive raptors by molecular methods in two rehabilitation facilities in North and North-east Iran and to define phylogenetic relationships of detected lineages circulating in raptors.

**Results:**

Molecular characterization of the haemosporidian parasite was accomplished by PCR-based method and DNA sequencing in 62 captive raptors. The overall prevalence was ~ 36% with higher infection of *Haemoproteus* spp. than *Leucocytozoon* spp. *Plasmodium* infection was not detected in any host. Results showed that 22 individuals (of 10 species) were infected with unique lineages. Genus *Haemoproteus* was detected in 26.66% of examined individuals (of eight species) and *Leucocytozoon* was found in 10% of individuals (of four species). The molecular analysis could detect ten lineages (nine *Haemoproteus* spp. and one *Leucocytozoon* spp.) which were categorizes as new and six lineages which have been previously detected in the other investigations.

**Conclusions:**

The Bayesian phylogenetic analysis derived from obtained data in the present study and published lineages in previous investigations indicated the probable host specificity of *Haemoproteus* and *Leucocytozoon* parasites in several sub-clades at hosts’ order and genus level. As monitoring the parasite loads of captive birds when admitted reduce the risk of infecting hosts in captivity at those locations, we designed this study to determine infection prevalence and genetic diversity of blood parasites in raptors examined in Iran. These results allow mapping of haemosporidian distribution and shed light on the depth of their diversity in Iran to protect species by identification of risk in rehabilitation facilities.

**Supplementary Information:**

The online version contains supplementary material available at 10.1186/s12862-022-02068-9.

## Background

Haemosporidian parasites (Apicomplexa: Haemosporida); *Haemoproteus, Plasmodium,* and *Leucocytozoon* species are cosmopolitan vector-borne organisms that are transmitted by biting hematophagous arthropods [1–7]. Haemoproteosis, avian malaria, and *Leucocytozoon*osis resulting from blood parasites may have great influences on their hosts’ life [2, 8–15]. Molecular characterization of haemosporidian parasites by using PCR-based methods has been extensively applied as quick, specific, and sensitive approaches in wildlife parasite screening and estimation of genetic diversity [16, 17]. Some genetic markers, such as the mitochondrial *cytb* gene have been barcoded more than 4100 distinct lineages in MalAvi [18]. These compiled datasets from various bird hosts around the world [2, 18–20] assign about 20% of molecular lineages to morphospecies [21]. Traditional microscopy method have described more than 250 haemosporidian morphospecies which can be applied as a complementary technique for screening parasites. The infection of each parasite is influenced by parasite strain, host species, and host-parasite-vector interactions in various geographical zones [22, 23]. Symptoms of haemosporidian infection may vary from no obvious clinical signs (asymptomatic) to severe signs; anemia, breath problems, thinness, lameness, poor appetite, and death [3, 14, 22, 24–27]. Sub-lethal influences of blood parasites in wild birds have not been completely understood. These parasites appeared harmless but some of these diverse intracellular parasitic species are the causative agents for serious avian diseases may promote clinical cases and mortality in non-adapted avian hosts [11, 28–30]. Consequently, these parasites pose a health concern, particularly in captive animals maintained at high densities in rehabilitation facilities, zoos, and gardens with imperfect resistance and increased stress due to human handling [31–34].

In this case, bird species that are under conservation concerns should be prioritized [35–39]. Although, birds have been transferred among zoological aviaries, rehabilitation centers, or zoos, there is no document on their health status or previous exposure to disease.. Thus, it is paramount to screening of blood parasites in captive hosts at admission to any rehabilitation facility, zoo, or gardens. Diagnosis of threatening factors such as infectious diseases indirectly plays a vital role in protecting endangered or threatened species by identification of risk factors that may boost the mortality or extinction caused by the reduced success of reproduction [40]. Monitoring the parasite loads of captive birds when admitted reduces the risk of infecting hosts in captivity at those locations [41–46]. No published investigation is available for the potential distribution of insect vectors of avian haemosporidian parasites in Iran. However, a low abundance of competent vectors, sampling time, and sample size may have influenced the low prevalence of detected parasites in the current study results. The small sample size was a restriction to defining host specificity. Bayesian analysis provide comparative results with published lineages in raptors that demonstrated the probable host specificity of several sub-clades at the order, family, and genus level. *Haemoproteus* and *Leucocytozoon* species clades displayed host specificity to some degree compared with *Plasmodium* spp.

Birds of prey are important in ecosystems and many studies have examined the genetic diversity, prevalence variability, host specificity, and description of new parasite lineages and species [40, 47–51]. Few investigations have targeted haemosporidian parasites in wild birds and captive hosts [12, 15, 52–57] to define the distribution and genetic diversity of parasites and vectors [12]. Because the identification of haemosporidia in the erythrocytic phase is required for conservation measures and protection of birds during rehabilitation programs [58], the present investigation aimed to determine the molecular prevalence of haemosporidian in captive raptors in two rehabilitation facilities and to assess the lineages diversity, and perceive the phylogenetic relationships using nested PCR amplification and DNA sequencing.

## Methods

### Blood samples collection and DNA extraction

This investigation was carried out by collecting blood samples from captive birds in two rehabilitation facilities in Khorasan Razavi and Golestan provinces, north and north-east of Iran, April to July 2015 and 2016 (Table 2). 50–100 µl of whole blood were taken from the brachial vein of sixty-two adult captive birds, which belong to three orders; Accipitriformes (~ 66%), Falconiformes (~ 26%), and Strigiformes (~ 8%) (Table 2), and to 13 species; *Accipiter badius* (n = 8)*, Aquila chrysaetos* (n = 2)*, Aquila rapax* (n = 5)*, Buteo buteo* (n = 21)*, Circus aeruginosus* (n = 2)*, Haliaeetus albicilla* (n = 1)*, Milvus migrans* (n = 1)*, Neophron percnopterus* (n = 1), *Falco subbuteo* (n = 1), *Falco tinnunculus* (n = 15), *Athene noctua* (n = 3), *Bubo bubo* (n = 1)*,* and *Otus scops* (n = 1). The examined species consist of 11 least concern species, one vulnerable (*Aquila rapax*) and one endangered species (*Neophron percnopterus*). Eight species are resident in Iran and the remaining are categorized as winter resident in some parts of country, migrants or summer breeders, shown in Table 2 [59]. Blood was preserved in Queen’s buffer for molecular experiments [60]. Genomic DNA was extracted using PrimePrep Genomic DNA Isolation Kit (GENETBIO Inc. Daejeon, South Korea) according to the manufacturer's instructions. The isolated DNA concentration and quality were estimated using a spectrophotometer (DeNovix Inc. USA).

### Nested PCR assay for parasites screening

A partial amplification of mt-DNA *cytb* gene (479 base pair) of the haemosporidian parasites was performed by nested PCR [16, 61]. The final volume of PCR reactions included 50 ng/µl of whole genomic DNA, 12.5 μl Ampliqon PCR master mix (AMPLIQON, Denmark), 0.6 mM of each primer and nuclease free water (up to 25 μl). To achieve the concise prevalence of haemosporidian parasites in raptors, we used two sets of primers. For the first PCR set, three parasitic genera were amplified by using general standard primers HaemNF1/HaemNR3 in the first reactions and HaemF/HaemR2 and HaemFL/HaemR2L in the second reactions (Table 1) [16, 61]. PCR cycling was performed as primary denaturation at 95 °C (5 min), annealing at 50 °C (30 s), extension at 72 °C (45 s), and finally followed by a final extension at 72 °C (10 min) which was run for 20 cycles in 1^st^ nested PCRs and 35 cycles in 2nd nested PCRs. Positive amplicons of the previous study and ultra-pure ddH_2_O were utilized as positive and negative controls for each PCR reaction set. PCR products were visualized on 2% agarose gel. Purification and sequencing were carried out using 20 μl of PCR products (479 bp) by Macrogene Co. (Seoul, South Korea). In addition, specific primers Plas1/HaemNR3 and 3760F/HaemJR4 for the detection of these parasites in raptors were used according to suggested procedures (Table 1) [47]. Purification and bi-directional sequencing of PCR products (524 bp) was performed by Codon Genetic Group (Tehran, Iran). Two set of primers were used for all host samples. The result showed that specific primers didn’t amplify all lineages which were detected by standard primers and vice versa; some of detected lineages were new.

**Table 1:** Primers used for nested PCR

Application	Primer (Forward/Reverse)	Sequence (5′–3′ direction)	Amplification round (nested PCR)	Target (genus)	References
General (all bird species)	HaemNF1 HaemNR3	CATATATTAAGAGAANTATGGAG TAGAAAGATAAGAAATACCATTC	Nest1	*Haemoproteus, Plasmodium, Leucocytozoon*	[61]
	HaemF HaemR2	ATGGTGCTTTCGATATATGCATG GCATTATCTGGATGTGATAATGGT	Nest 2	*Haemoproteus, Plasmodium*	
	HaemFL HaemR2L	ATGGTGTTTTAGATACTTACATT CATTATCTGGATGAGATAATGGIG	Nest 2	*Leucocytozoon*	[16]
Specific (raptors)	Plas1 HaemNR3	GAGAATTATGGAGTGGATGGTG ATAGAAAGATAAGAAATACCATTC	Nest 1	*Haemoproteus, Plasmodium, Leucocytozoon*	[47]
	3760F HaemJR4	GAGTGGATGGTGTTTTAGAT GAAATACCATTCTGGAACAATATG	Nest 2	*Haemoproteus, Plasmodium, Leucocytozoon*	

### DNA sequencing and phylogenetic analysis

The novelty of amplified sequences’ was confirmed by “Nucleotide BLAST” in National Center for Biotechnology Information (NCBI) (Basic Local Alignment Search Tool) search [24] and MalAvi blast hits [18]. Mitochondrial lineage with one or more substitutions was considered a new lineage [16], [16]. The amplified sequences were named based on MalAvi nomenclature [18]. Raw sequences were edited and aligned using Bioedit [63] and online MAFFT programs [64]. Three sequences from two individuals of *Buteo buteo* and two *Aquila rapax*, and one *Athene noctua* who had mature *Haemoproteus* species gametocytes in their blood smears, were excluded from the analysis because of having double peaks in the electropherograms.

**Fig. 1:**
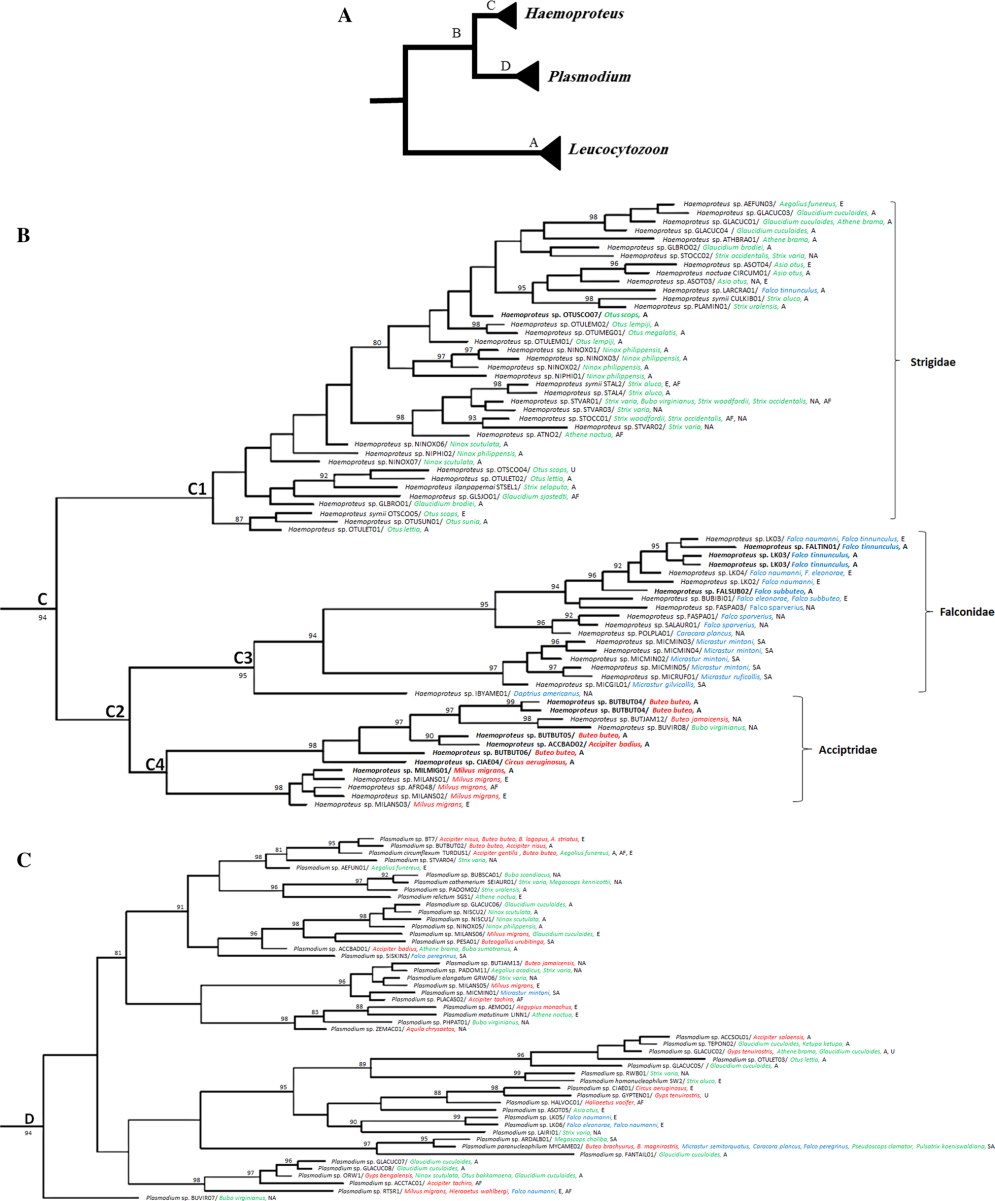

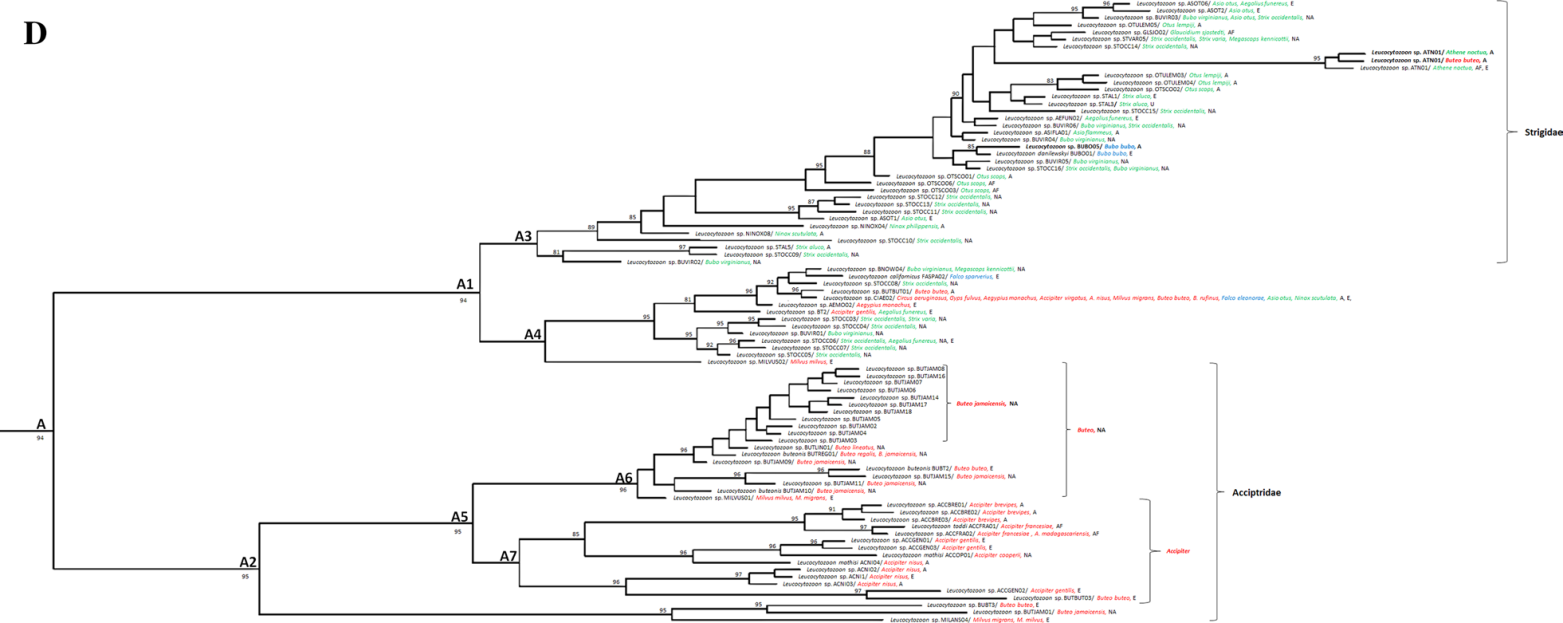
Bayesian analysis of haemosporidian *cytb* lineages in raptors data deposited in MalAvi, GenBank and present study sequences. Posterior probability of Bayesian analysis are specified for each branch (> 80). Detected lineages of the current investigation are in bold. Host species of Accipitriformes, Falconiformes, and Strigiformes are specified with red, blue, and green colors. Schematic image of entire tree which is separated to each genus (**a**), *Haemoproteus* spp. (**b**), *Plasmodium* spp. (**c**), *Leucocytozoon* spp. (**d**)

**Fig. 2:**
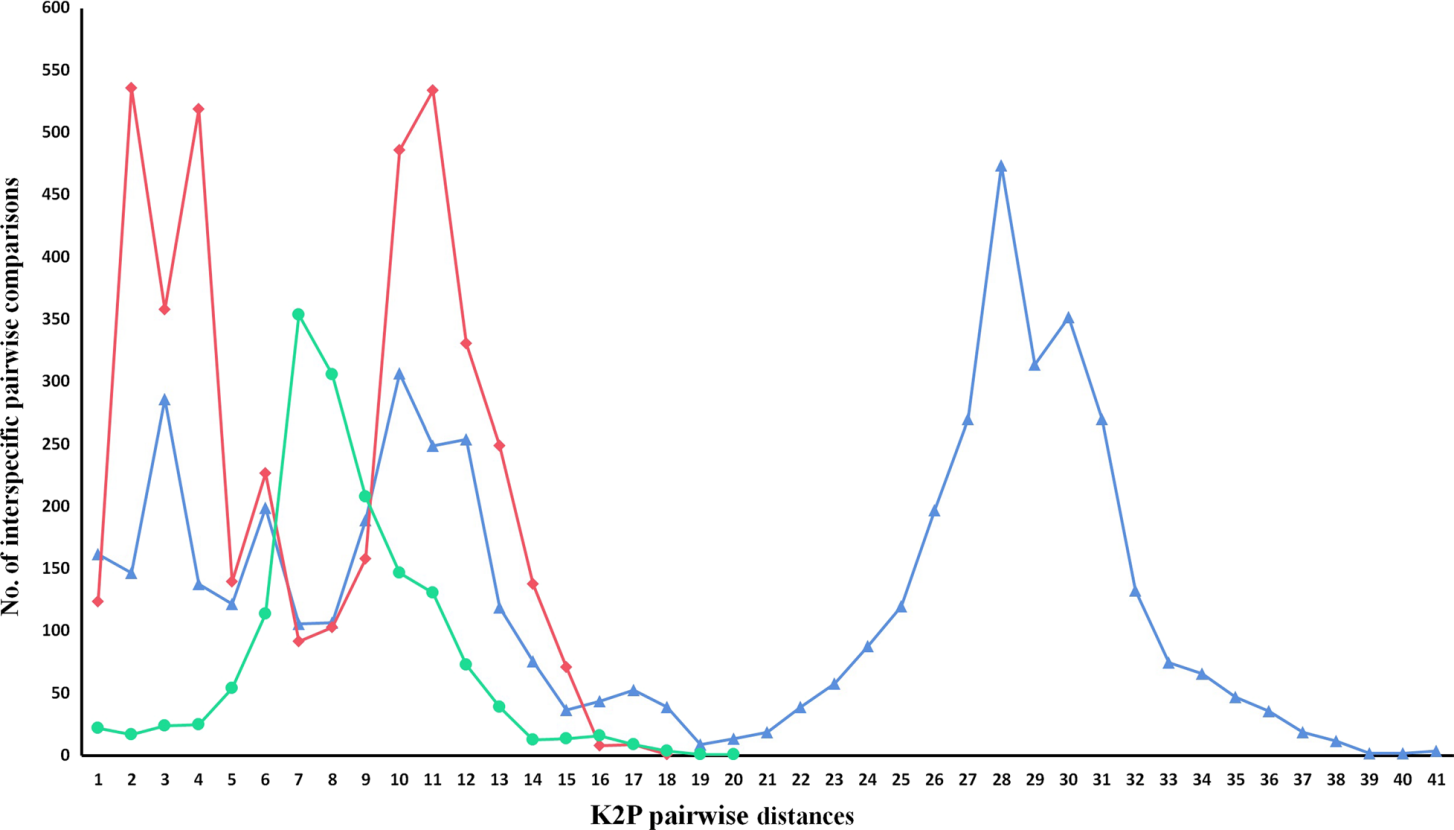
Comparison of interspecific pairwise Kimura 2-parameter (K2P) distances for haemosporidian parasitic genus detected in raptors. *Haemoproteus* (red), *Plasmodium* (green), and *Leucocytozoon* (blue) are specified

To perceive the phylogenetic relationship of haemosporidian parasites with previously described lineages circulating in raptors, a dataset containing our sequences and those present on the online database MalAvi was constructed to insert our sequences in a wider context (sequences of similar hosts’ haemosporidian sequences were retrieved) (Fig. 1). A table containing the general information of the sequences belonging to the MalAvi dataset is given in Additional File 1. The sequences divergence of blood parasite lineages was measured with the Kimura 2-parameter (K2P) distance matrix, implemented in MEGA6.0 using the pairwise comparison of all sequences [65] and interspecific pairwise distances were calculated by converter program, Excalibar v.1.1, is shown in Fig. 2 and Table 4 [66].

Barcoding gap reconstruction for each parasite genus was performed by ABGD analysis [67] run online (https://bioinfo.mnhn.fr/abi/public/abgd/abgdweb.html). This method finds a range of previous intraspecific distances (calculated from Pmin to Pmax, with P Steps). A proxy for the minimum relative gap width is shown by the X value (relative width). The selected distance calculation method was Kimura (K80) TS/TV and relative width = 1 for *Haemoproteus* and *Leucocytozoon* and relative width = 0.75 for *Plasmodium*. The other variables were based on default settings (Additional file 2 and Additional file 3).

Phylogenetic analyses were performed by Bayesian inference executed in MrBayes v3.2 software [68] with two concurrent MCMC simulations for 10,000,000 generations and sampled every 1000 generations. The posterior probabilities were measured at the end of the analysis when the burn-in period of 50% was set and the chains reached stationary status. ModelTest v.7 software was used for the best evolutionary model selection (GTR + I + G) [69]. The resultant phylogenetic tree is visualized by FigTree v1.4 [70]. Moreover, the analysis of Poisson Tree Processes (PTP) was performed to reveal the species delimitation [71] on the online server https://species.h-its.org/. PTP algorithm uses the number of mutations (inferred from branch lengths) for speciation modeling on branches. The resultant tree showed the position of lineages at the genus level (Additional file 4).

## Results

### Prevalence of haemosporidian parasites and genetic diversity of identified lineages

The overall prevalence of detected haemosporidian parasites by using nested PCR assay was 36.66% (95% CI 24.11, 49.22) in comparison with the reported prevalence of 28.33% (95% CI 16.59, 40.07) by microscopic examinations in our former study (Table 2). The molecular method revealed that 22 individuals of each ten species were infected with one haemosporidian lineage. Compared results of prevalence for genus and identification method are presented as follows: morphological method; *Haemoproteus* spp. 25% (95% CI 13.72, 36.28), *Leucocytozoon* spp. 3.33% (95% CI 1.32, 8.01) and molecular method; *Haemoproteus* spp. 26.66% (95% CI 15.15, 38.19), *Leucocytozoon* spp. 10% (95% CI 2.18, 17.82). Microscopic results have shown that 15 individuals (24.1%) were infected by *Haemoproteus* spp. and two individuals were infected (3.22%) by *Leucocytozoon* spp. The obtained result was reported at the genus level. The compared results of microscopic [52] and molecular methods presented in this study revealed that the molecular method could detect more infections rather than morphological examination but failed to amplify five morphologically positive parasites which may be co-infected. Regardless of amplification during PCR, the blast hit showed no results, and the presence of double peaks seen in sequences enabled us to omit them from analysis and prevalence calculation.

**Table 2:** *Haemoproteus* and *Leucocytozoon* lineages isolated from raptors in rehabilitation facilities in Iran

Host order	Host species	Common name	Host residency (Iran)	Sampling sites (n)	Mic. Hae.^1^	*Haemoproteus* lineages (no.)	Mic. Leu.^1^	*Leucocytozoon* lineages (no.)
Accipitriformes	*Accipiter badius*	Shikra	NA	R (8)	1	**hACCBAD02 (1)**	0	–
	*Aquila chrysaetos*	Golden eagle	+	R (1), G (1)	0	–	0	–
	*Aquila rapax*	Tawny eagle	+	R (1), G (4)	2	–	0	–
	*Buteo buteo*	Eurasian buzzard	+	R (15), G (6)	5	**hBUTBUT04 (2)**^**2**^ **hBUTBUT05** (1) **hBUTBUT06** (1)	0	lATN01 (1) lMULVIS01 (1) lBUBT03 (1)^2^
	*Circus aeruginosus*	Western marsh harrier	+	G (2)	1	**hCIAE04** (1)	0	–
	*Haliaeetus albicilla*	White-tailed eagle	+	G (1)	0	–	0	lCIAE02 (1)
	*Milvus migrans*	Black kite	NA	G (1)	1	**hMILMIG01** (1)	0	–
	*Neophron percnopterus*	Egyptian vulture	NA	G (1)	0	–	0	–
Falconiformes	*Falco tinnuculus*	Common kestrel	+	R (12), G (3)	3	**hFALTIN01** (1) hLK03 (4)	0	–
	*Falco subbuteo*	Eurasian hobby	NA	R (1)	1	**hFALSUB02** (1)	0	–
Strigiformes	*Athene noctua*	Little owl	+	R (3)	0	hTYTAL04 (1) hLK03 (1)	1	lATN01 (1)
	*Bubo bubo*	Eurasian eagle-owl	+	R (1)	0	–	1	**lBUBO05 (1)**
	*Otus scops*	Eurasian scops owl	NA	R (1)	1	**hOTSCO07** (1)	0	–
Total (percentage)				**62**	**15 (24.1%)**	**16 (25.8%)**	**2 (3.22%)**	**6 (9.67%)**

Rehabilitation facilities are in Razavi Khorasan (R), Golestan (G) provinces. Novel lineages are specified in bold

^1^Number of positive samples by microscopic examination are retrieved from [52]; Mic. Hae: number of *Haemoproteus* positive samples, Mic. Leu: number of *Leucocytozoon* positive samples. The detected parasites by specific primers are underlined

^2^Co-infection

All bird hosts, except for the golden eagle (*Aquila chrysaetos*), tawny eagle (*Aquila rapax*), and Egyptian vulture (*Neophron percnopterus*) were parasitized by haemosporidian lineages (Table 2). 16 of 62 individuals were positive in the nested-PCR test for *Haemoproteus* spp., and six samples were positive targeting *Leucocytozoon* parasite infections. One Eurasian buzzard individual (*Buteo buteo*) was co-infected by *Haemoproteus* and *Leucocytozoon* species. *Plasmodium* parasite infection was not observed in morphological and molecular examinations. 14 identified infected individuals sampled from a rehabilitation facility in Khorasan Razavi (one co-infected sample) and eight individuals from Golestan province centers. The distribution of previously detected lineages in similar hosts examined in this study and other countries is summarized in Table 3. The compared data showed that most infections in other countries were from *Leucocytozoon* spp. *Buteo buteo* and *Milvus migrans* have shown the most variety of infecting lineages. Similar to our investigation, no haemosporidian lineage has been discovered in *Neophron percnopterus* and *Aquila rapax* around the world (Table 3).

**Table 3:** Contribution of known haemosporidian lineages in raptors

	*Accipiter badius*	*Aquila chrysaetos*	*Aquila rapax*	*Buteo buteo*	*Circus aeruginosus*	*Haliaeetus albicilla*	*Milvus migrans*	*Neophron percnopterus*	*Falco tinnunculus*	*Falco subbuteo*	*Athene noctua*	*Bubo bubo*	*Otus scops*
**hACCBAD02**	1												
hATN02											1		
hBUBBUB01												1	
hBUBBUB02											1	1	
hBUBBUB03												1	
hBUBIBI01									1	1	1	1	
**hBUTBUT04**				2									
**hBUTBUT05**				1									
**hBUTBUT06**				1									
**hCIAE04**					1								
hCIRCUM01											1		
hCULKIB01												1	
hFALAMU01											1		
hFALSUB01										1			
**hFALSUB02**										1			
**hFALTIN01**									2				
hFALTIN02									1				
hFALTIN03									1				
hFALTIN04									1				
hFALTIN06									1				
hFALTIN07									1				
hFALTIN08									1				
hFALTIN09									1				
hLARCRA01									1				
**hLK03**									5		1		1
hMILANS01							1						
hMILANS02							1						
hMILANS03							1						
**hMILMIG01**							1						
hOTSCO05													1
**hOTSCO07**													1
hOTUSCO01									1				1
hOTUSCO02													1
**hTYTAL04**											1		
lANACU04									1				
lASOT06									1				
**lATN01**				1							2		
lBUBO01												1	
**lBUBO05**												1	
lBUBT2				1									
**lBUBT3**				2									
lBUTBUT01				1									
lBUTBUT03				1									
**lCIAE02**				2	1	1	3					1	
lFALTIN10									1				
lFALTIN11									1				1
lFALTIN12									1				
lMILANS04							1						
lMILVUS01							1						
lOTSCO01												1	1
lOTSCO02													1
lOTSCO03													1
lOTSCO06													1
lOTUSCO03													1
lOTUSCO04													1
lOTUSCO05													1
lOTUSCO06													1
lOTUSCO07													1
lOTUSCO08													1
lOTUSCO09													1
pACCBAD01	1												
pALARV04												1	
pBT7				1									
pBUTBUT02				1									
pCIAE01					1								
pEMSPO06													1
pFALTIN05									1				
pFALTIN14									1				
pFALTIN15									1				
pGRW11											1		
pLINN1											1		
pMILANS05							1						
pMILANS06							1						
pORW1													1
pOTUSCO10													1
pRTSR1							1						
pSGS1											3		
pTURDUS1				2									
pZEMAC01		1											

Detected lineages in the current investigation are specified in bold

Detected *Haemoproteus* spp. lineages include hACCBAD02, hBUTBUT04, hBUTBUT05, hBUTBUT06, hCIAE04, hMILMIG01, hFALTIN01, hLK03, hFALSUB02, hTYTAL04, and hOTSCO07. In addition to, the identified *Leucocytozoon* spp. lineages consist of lATN01, lMULVIS01, lBUBT03, lCIAE02, and lBUBO05. *Haemoproteus* spp. lineages were detected from eight bird species; *Accipiter badius, Buteo buteo, Circus aeruginosus, Milvus migrans, Falco tinnunculus, Falco subbuteo, Athene noctua,* and *Otus scops,* and *Leucocytozoon* spp. lineages were discovered in four raptors species; *Buteo buteo, Haliaeetus albicilla*, *Athene noctua,* and *Bubo bubo.* New lineages were detected in Shikra (hACCBAD02), Eurasian buzzards (hBUTBUT04, hBUTBUT05, hBUTBUT06), Western marsh harrier (hCIAE04), Black kite (hMILMIG01), Common kestrel (hFALTIN01), Eurasian hobby (hFALSUB02), Eurasian scops owl (hOTSCO07), and Eurasian eagle owl (lBUBO05). Common kestrel, Little owl, Eurasian buzzard, Little owl, Eurasian buzzard, and White-tailed eagle parasitized by lineages hLK03, hTYTAL04, lATN01, lBUBT03, lMULVIS01, and lCIAE02, and were introduced as new host records examined in Iran (Scientific names of infected hosts are given in Table 2). Lineage hBUTBUT04 was found in two individuals of the same host species, and lATN01 (Accipitriformes and Strigiformes) and hLK03 (Falconiformes and Strigiformes) were discovered in two different orders (Table 2). Furthermore, using molecular approach revealed the co-infection of one *Buteo buteo* by two lineages; hBUTBUT04 and lBUBT03 (Table 4).

**Table 4:** Genetic information for haemosporidian parasite’ sequences detected in raptors and the retrieved data from MalAvi

Genus/ sequences information	No. of sequences	Conserved site (n)	Variable site (n)	Parsimony informative site (n)	Singleton site (n)	T (%)	C (%)	A (%)	G (%)	No. of interspecific pairwise comparisons	K2P genetic distances (%)	Mean of K2P genetic distances (%)
*Haemoproteus*	91	**351**	128	97	31	**44.4**	13.2	28.6	**13.8**	**5250**	0–16.7%	6.45%
*Leucocytozoon*	**103**	260	**219**	**190**	29	42.2	**14.6**	29.3	**13.8**	4085	**0–40.4%**	**17.42%**
*Plasmodium*	58	334	145	114	**31**	43.7	13.2	**29.5**	13.6	1653	0–23.1%	6.69%

The highest value in each column is specified in bold

Number of sequences, conserved, variable, parsimony, and informative site, singleton sites, percentage of each nucleotide and number of interspecific pairwise comparisons for each genus are given

### Interspecific distances and phylogeny of known haemosporidian lineages in raptors

The Bayesian phylogenetic tree was reconstructed by the present data detected in this investigation and deposited sequences of all lineages that infect raptors in MalAvi (n = 249). The results demonstrated the placement on three distinctive clades; A (*Leucocytozoon* spp.), D (*Plasmodium* spp.), and C (*Haemoproteus* spp.) (Fig. 1). The available data of infection in raptors around the world included sequences retrieved from wild (87%) and captive individuals (13%) in Asia (49%), Europe (22.3%), South America (8.7%), North America (15.3%), Africa (3.9%), and unknown places (0.9%). Average K2P distance was calculated to show whether there is a sequence variability for each parasitic genus. These results showed different genetic variations for each genus (Fig. 2). The highest and lowest interspecific distances range belonged to *Leucocytozoon* spp. (0–40.4%) and *Haemoproteus* spp. (0–16.7%). The maximum and the minimum mean of K2P genetic distances (%) was calculated to *Leucocytozoon* spp. (17.42%) and *Haemoproteus* spp. (6.45%). The number of conserved, variable, parsimony, and singleton sites is mentioned in Table 3. The highest number of conserved sites (73.4%) belonged to *Haemoproteus* and *Leucocytozoon* had the highest proportion of variable sites (45.81%), parsimony informative sites (39.74%), and GC content (28.4%). The maximum number of singleton sites belonging genus *Plasmodium* (6.48%) (Table 3). In the Bayesian phylogenetic tree, some *Leucocytozoon* spp. sequences are placed within host order-specific sub-clades. Except for some lineages, A2 is Accipitriformes-specific and A3 is Strigiformes-specific. Within A5, all but one raptor parasite fall into *Accipiter*-specific and *Buteo*-specific monophyletic sub-clades. Returning to sub-clade A6, all lineages are parasites of *Buteo* except MILVUS01 detected in *Milvus milvus* and *M. migrans* from Europe. Disregarding the placement of BUTBUT03 and BUBT3 detected in *Buteo buteo*, the remaining raptor parasite lineages fall into *Accipiter* sub-clade A7. Within A1, excluding several lineages, remaining lineages in A3 are positioned as Strigiformes-specific sub-clade while the other sub-clade (A4) includes parasites from three raptors orders. *Plasmodium* spp. clade is sister taxon to *Haemoproteus* spp. clade (clade C) and contains parasites associated with three raptors orders. Except for pMYCAME02 and pRTSR1, the remaining *Plasmodium* lineages are discovered in one order (clade D). Within *Haemoproteus* spp., raptor parasites are monophyletic and consisted of two sub-clades, C1 (C2; Falconiformes-specific and C3; Accipitriformes-specific) and C4 (Strigiformes-specific, except for several lineages). Most of the detected lineages of this study are grouped within the C2 sub-clade (Fig. 1a–d). Sub-clade C4 and A1 comprise exclusively Strigiformes parasites (with some exceptions) while clade A2 and C3 contain parasites hosted by Accipitriformes and A6 consist of *Buteo* parasites from North American and *Accipiter*. In addition, the sub-clade of A1 (*Leucocytozoon* spp.) and clade D (*Plasmodium* spp.) are included more generalist lineages (Fig. 1a–d).

The position of lineages in the maximum likelihood (ML) tree showed the similarity of sub-clades and also some differences at the genus level. In comparison with the Bayesian tree where *Haemoproteus* and *Plasmodium* lineages were positioned as sister clades and *Leucocytozoon* was situated in a basal out-group, the location of lineages in the ML tree was different and three parasite genera were placed in a paraphyletic manner (Additional file 4). So, for the precise interpretation of species delimitation, using an extensive dataset is essential and the analysis should be concluded more cautiously by different algorithms.

## Discussion

In the current investigation, the molecular characterization of haemosporidian parasites infecting ten species of captive birds was established in two rehabilitation facilities in Iran. Several raptors were new hosts from this region required such investigations to clarify the diversity of blood parasite species and their hosts through conservation programs. Numerous studies have reported the high prevalence of haemosporidian parasite infections in captive birds leading to immense mortality than in wild hosts [72, 73]. Investigating birds in captivity provides an opportunity for researchers and students to explore the maximum host range of parasite lineages which are very hard to sample in nature [74]. Birds in captivity are not in a natural environment and may have less resistance to parasitic agents in a situation imposing increased stress. Such investigations may provide more information about health status, deterioration factors, and death in captive birds referable wild hosts [75, 76]. Various haemosporidian parasites infect raptors, especially captive hosts who may be more exposed. The captivity situations may lead to infection outbreaks in domestic and wild hosts [32, 77]. Improved and periodic screening, molecular epidemiologic investigations, and control of possible vectors in managerial programs from a conservation viewpoint are recommended as essential procedures to protect valuable raptor hosts. Lack of suitable foods and stressful conditions can significantly increase infection prevalence and decrease the immune responses [73, 77] that highlight the requirement of protecting wild hosts that may have interacted with captive birds or be infected by potential vectors in adjacent locations. These unusual interactions may cause the transmission of parasites into new wild or migrant birds, such as the mortality that occurred in captive psittacines parrots, *Cyanoramphus auriceps, Bolborhynchus lineola,* and *Melopsittacus undulates* in two different aviaries from Switzerland and Germany which showed *Haemoproteus* infections [78, 79]. The mortality of Egyptian geese (*Alopochen aegyptiaca*) in São Paulo zoo and European parrots were attributed to *P. nucleophilum* and *H. minutus* infections, respectively [77, 80]. Histopathological and molecular approaches documented the presence of *Plasmodium* spp., as a causative agent for mortality in raptor species at a safari park in Italy [73]. A recorded prevalence of 12.6% in 677 hosts sampled in the São Paulo zoo, showed infection to 14 lineages of *Plasmodium* spp. and 2 *Haemoproteus* spp. of which 8 were novel [62]. Wild perching birds have been considered as potentially infected hosts for transmission of infection to captive birds by vectors’ biting [81]. The high rate of mortality in captive Magellanic penguins (*Spheniscus magellanicus*) in a zoo in Southern Brazil was confirmed by *Plasmodium* parasite infections (pTURALB01), a previously detected lineage in a passerine host, *Turdus albicollis* [82]. Moreover, migratory species are acknowledged frequently as active carriers of various haemosporidian lineages to infect captive and/or non-migrant species [83].

The infection prevalence and genetic diversity of haemoparasites of captive raptors examined in Iran have not been studied in detail. In present study, ten new lineages and six previously known *Haemoproteus* spp. and *Leucocytozoon* spp. lineages are detected in examined hosts from this region. In the current study, no captured bird was infected by *Plasmodium* spp. Frequent elements may be related to infection prevalence of haemosporidian in birds, encompassing host-related features; species, sex, host-vector-parasite relationships, immune system, ecological factors; study site, habitats, sampling time, detection procedures; and vector’s related characteristics; host specificity and vector competency [2, 20, 50, 72, 84]. PCR-based identification method revealed *Haemoproteus* and *Leucocytozoon* of ~ 36% of examined individuals. The highest infection of *Haemoproteus* spp. was found in Accipitriformes, and *Buteo buteo* had the highest number of captured individuals in both rehabilitation facilities. These results are consistent with previous investigations in Iran that have shown a high diversity of *Haemoproteus* spp. lineages in detected hemoparasites [52–56]. The higher prevalence of *Haemoproteus* spp. (78%) in comparison with *Leucocytozoon* spp. (35%) in 55 captive raptors in another part of the world was consistent with our investigation [85]. Both a very high overall prevalence of 97.6% (genus *Plasmodium*) and a very low prevalence of 2.4% (genus *Haemoproteus*) have been recorded in captive birds in Brazil [62]. Application of PCR-based methods for detection of blood haemosporidian parasites in 167 individuals of owls belonging to 12 species from Thailand showed an overall prevalence of 34.1% and reported 17 new detected lineages [58]. Their findings may indicate high transmission of *Haemoproteus* and *Plasmodium* species in owls in comparison with previous studies in Asia, Korea (62.8%) [86], and Japan (57.1%) [87]. The low prevalence of blood parasites (16.7%) in a collection of 324 *Falco eleonorae* nestling and adult individuals highlighted the possible effect of ecological factors such as occupied habitats, host-vector-parasite relationship, and hosts immune system efficacy [50].

Among Accipitriformes, *Accipiter badius* has not been previously identified as a *Haemoproteus* novel host, except for an unpublished record in MalAvi in which pACCBAD01 was recorded from Thailand (Salakij et al. unpubl, recorded in MalAvi). *Haemoproteus* spp. lineage hACCBAD02 isolated from *Accipiter badius,* displayed 99% similarity as opposed to the nearest lineage hARBRU02. *Circus aeruginosus* as a new host examined in Iran for *Haemoproteus* spp. join records of this species parasitized by pCIAE01 and lCIAE02 in Germany [88]. Novel lineage hCIAE04 detected in *Circus aeruginosus* demonstrated 99% similarity with hPLONEL01 recorded in the MalAvi database. In addition, *Haliaeetus albicilla* is a new host for lCIAE02 detected in birds examined in Iran. Lineage hLK03 as the reference lineage for hFALTIN01 and hFALSUB02 has been detected in *Falco naumanni* and *F. tinnunculus* from Spain and Germany [88, 89]. Although there have been limited studies on blood parasites of raptors, the lineage of hLK03 has not yet been found in other avian families. Our results presented the infection to hLK03 in *F. tinnunculus* (Falconiformes) and *Athene noctua*, as a new order member infection (Strigiformes). Lineage hTYTAL04 detected in *Athene noctua,* is reported as another new host from this region. We isolated *Haemoproteus* spp. hBUTBUT04 from two individuals of *Buteo buteo*; this lineage shows 99% similarity with hSISKIN1 has been reported in various species of Passeriformes [90, 91] and Piciformes [56]. The newly discovered lineage hBUTBUT06 was 99% identical to the reference sequence hPIRUB01 has been previously recorded in *Pitangus sulphuratus* from Brazil [92]. The other new *Haemoproteus* spp. lineage hBUTBUT05 discovered from *Buteo buteo,* showed 99% similarity in comparison with hARBRU02 was recovered in *Buarremon brunneinucha* from South America [93, 94]. *Haemoproteus* spp. lineage hCELEC01 was the nearest lineage to newly isolated hOTSCO07 in *Otus scops* (99% similarity) had been registered on the MalAvi online database. *Haemoproteus* spp. lineage hMILMIG01 found in *Milvus migrans* in the present study was similar to hMILANS03 (99% similarity) was isolated from the same host species in Spain [47]. The shared lineage lATN01 was discovered in *Athene noctua* and *Buteo buteo* belonging to two different orders examined in Iran. This lineage was previously recovered in *Athene noctua* on other continents from Morocco and Portugal [95]. However, lineages lMULVIS01 and lBUBT03 detected in *Buteo buteo* join records of *Leucocytozoon* spp. in this species.

From a conservation perspective, the discovery of co-infections is related to virulence [96] and there are some limitations in the identification of *Plasmodium* and *Haemoproteus* species by PCR-based methods, therefore microscopic method can serve as a diagnostic method [97]. In the current study, we focused on molecular results, and our morphological examination results have confirmed the parasite infection at the genus level [52].However, we used two sets of primers to identify general and specific lineages for raptors ambiguous sequences with double peaks, due to un-successful amplification of parasite or co-infections within the same host, necessitate the re-examination by other diagnostic tools in further investigation. Molecular diagnostic methods have been established comparatively as applicable as traditional techniques using light microscopy for haemosporidian parasites detection [16, 78, 96, 98]. On the other side, morphological procedures may even undervalue low parasitemia, abortive infections (unsuccessful development of the parasite in which gametocytes are not in smears), or cryptic species, when compared with molecular methods are known for underestimation co-infections [97, 99]. As the detection of simultaneous infections is frequently common in avian hosts [100], routine molecular methods not able to validate co-infections. The microscopic method can identify co-infections. Therefore, a combination of molecular and microscopic methodologies increases the probability of discovery of haemosporidian infections at the species level in wild birds [101, 102]. Besides, the parallel application of diverse primers [96, 103], multiplex PCR [9], RT-PCR [10, 33], high-resolution melt (HRM) analyses [23], and next-generation sequencing (NGS) are recommended to increase the sensitivity of parasite diversity in haemosporidian detection [7]. Nonetheless, these approaches are expensive and time-consuming and may not be applicable in every geographical region in surveillance investigations.

The significant finding of the current investigation was the molecular screening of avian haemosporidian blood parasites in captive raptors maintained in two rehabilitation facilities and documentation of novel information on the genetic diversity of haemosporidian lineages in an unexamined geographical region in west Asia. As the monitoring parasite loads of captive birds when admitted reduce the risk of infecting hosts in captivity at those locations, we designed this study to determine blood parasites prevalence and genetic diversity in raptors examined in Iran. The results allow mapping of haemosporidian distribution and shed light on the depth of their diversity in Iran to protect species by identification of risk in rehabilitation facilities.

## Supplementary Information

**Additional file 1. **The general information of the sequences retrieved from the MalAvi dataset. This table includes the lineages’ names, genus, host family, host species, host statues, continent, country, and references.

**Additional file 2. **Barcoding gap reconstruction for each parasite genus is done by ABGD analysis. The resultant histograms are shown for *Haemoproteus, Leucocytozoon*, and *Plasmodium*. These graphs illustrate the genetic distances (X) and the number ofinterspecific pairwise comparisons (Y).

**Additional file 3. **More detailed information about the number sequences for each genus analysis, the number of partitions, and the frequency of groups dedicated to each partition are given.

**Additional file 4. **The Poisson Tree Processes (PTP) was performed to reconstruct maximum likelihood (ML) tree analysis on the online server https://species.h-its.org/. The resultant tree showed the position of lineages at the genus level.

## Data Availability

Amplified sequences were submitted in the GenBank under accession numbers MN224217-MN224231 and MW209702-MW209713.
