# Supplementary figures and images for: Prevalence of co-infection and genetic diversity of avian haemosporidian parasites in two rehabilitation facilities in Iran: implications for the conservation of captive raptors
Source: BMC Ecol Evol. 2022 Oct 8;22:114. doi: 10.1186/s12862-022-02068-9 (PMC9547439; doi:10.1186/s12862-022-02068-9)

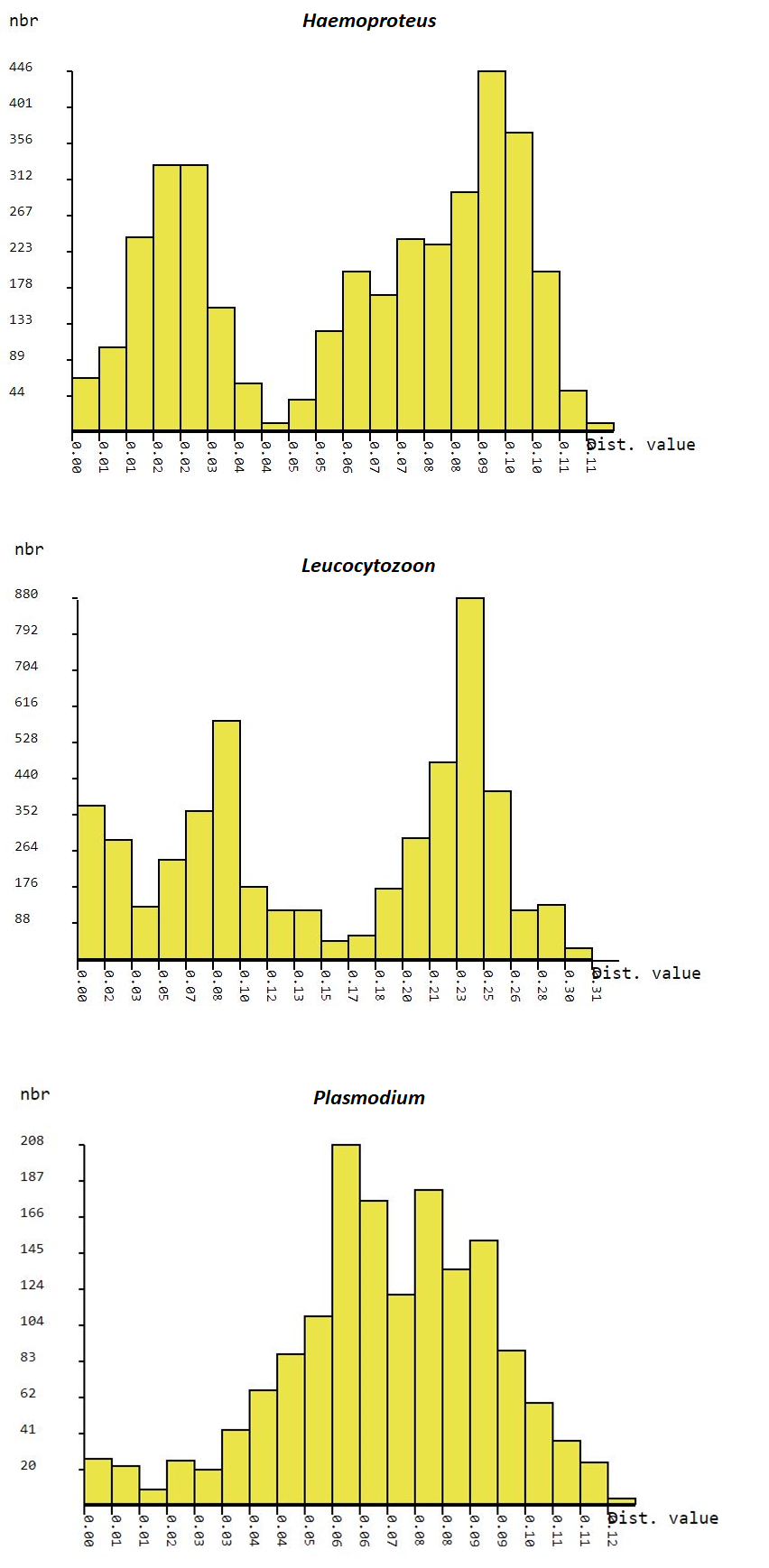

Supplement: Supplementary file 2 — Additional file 2. Barcoding gap reconstruction for each parasite genus is done by ABGD analysis. The resultant histograms are shown for Haemoproteus, Leucocytozoon, and Plasmodium. These graphs illustrate the genetic distances (X) and the number ofinterspecific pairwise comparisons (Y). [file 12862_2022_2068_MOESM2_ESM.tif]
